# Calculated plasma volume status in hemodialysis patients

**DOI:** 10.1080/0886022X.2024.2322685

**Published:** 2024-02-27

**Authors:** Qiankun Zhang, Hang Fang, Lie Jin

**Affiliations:** a Division of Nephrology, the Fifth Affiliated Hospital of Wenzhou Medical University, Lishui Central Hospital, Lishui Hospital of Zhejiang University, Lishui, China; b Division of Nephrology, Quzhou People’s Hospital, Quzhou, China

**Keywords:** Calculated plasma volume, hemodialysis, bioelectrical impedance analysis, volume overload, all-cause mortality

## Abstract

**Background:**

Plasma volume (PV) calculated from hematocrit and body weight has applications in cardiovascular disease. The current study investigated the validity of the calculated PV for predicting volume overload and its prognostic utility in patients undergoing hemodialysis (HD).

**Patients and methods:**

Fifty-four HD patients were prospectively enrolled, and their actual PV (aPV) and relative PV status (PVS) were calculated. Bioelectrical impedance analysis (BIA) with assessment of and total body water (TBW), intracellular water (ICW), extracellular water (ECW), and overhydration (OH) and routine blood examinations were performed before dialysis. A second cohort of 164 HD patients was retrospectively enrolled to evaluate the relationship between the calculated PVS and the outcome, with an endpoint of all-cause mortality.

**Results:**

aPV was significantly associated with TBW, ICW, ECW, OH, and ECW/TBW (all *p* < 0.001), and most strongly with ECW (*r* = 0.83). aPV predicted the extent of volume overload with an AUC of 0.770 (*p* < 0.001), but PVS did not (AUC = 0.617, *p* = 0.091). Median follow-up time was 53 months, during the course of which 60 (36.58%) patients died. Values for PVS (12.94 ± 10.87% vs. 7.45 ± 5.90%, *p* = 0.024) and time-averaged PVS (12.83 ± 11.20 vs. 6.78 ± 6.22%, *p* < 0.001) were significantly increased in patients who died relative to those who survived. A value of time-averaged PVS >8.72% was significantly associated with an increased incidence of all-cause mortality (HR = 2.48, *p* = 0.0023).

**Conclusions:**

aPV was most strongly associated with ECW measured using BIA. HD patients with higher time-averaged PVS had a higher rate of all-cause mortality.

## 1. Introduction

Volume overload in hemodialysis (HD) patients is a major risk factor for all-cause and cardiovascular mortalities [1,2]. Managing body volume in patients requiring maintenance HD remains a critical challenge because of the paucity of convenient and validated measurement techniques [3]. The accurate and objective diagnosis of subclinical volume overload has been the subject of intensive research over the last 50 years [4]. Bioelectrical impedance analysis (BIA) is a promising tool that allows quantification of body composition, including values for intracellular water (ICW) and extracellular water (ECW). Volume overload assessed by BIA has been linked to the decreased overall survival of HD patients [5,6], but the methodology lacks the capacity to estimate interstitial and intravascular fluid compartments and is expensive and inconvenient [7,8]. Thus, great clinical value is attached to the development of new methods to diagnose volume overload in patients undergoing HD.

Several factors such as ejection fraction, salt intake, and hematocrit (Hct) may affect volume calculations. A novel technique for the calculation of plasma volume (PV) Hct and body weight (BW) values has been used in patients with chronic heart failure (CHF). Ling et al. evaluated the agreement between the calculated actual PV (aPV) and aPV measured by the gold standard ^125^Iodine-human serum albumin method in 2015. A good correlation was found between the calculated and measured aPV values in 119 normal subjects and 30 patients with CHF [9]. In addition, relative PV status (PVS) is associated with mortality and first morbid events in patients [9]. Thereafter, the prognostic implications of PVS in patients with CHF have been demonstrated [10–12]. Moreover, PVS has prognostic value for patients undergoing coronary bypass graft surgery, transcatheter aortic valve replacement, and elective percutaneous coronary intervention [13–15].

However, no data are available regarding the utility of the calculated PV in HD patients. The current study investigated the validity of the calculated PV for assessing volume overload in patients on HD and its prognostic utility.

## 2. Patients and methods

**Figure 1.**
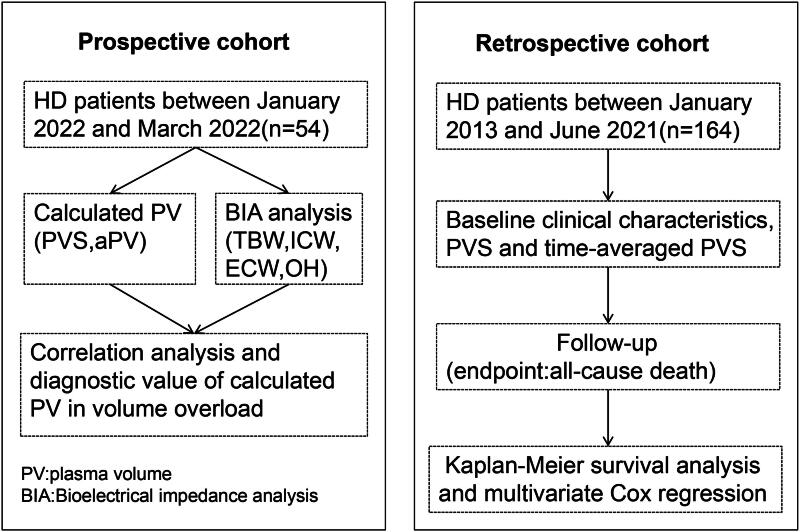
Flow chart illustrating the protocol.

The study was divided into two cohorts: a prospective cohort, focusing on the correlation between PV and BIA, and a retrospective cohort, examining the relationship between calculated PVS and outcomes. The study’s process flow is illustrated in Figure 1.

### 2.1. Prospective cohort

#### 2.1.1. Patients enrollment

HD patients were prospectively enrolled from the Lishui Central Hospital between January 2022 and March 2022. The inclusion criteria were as follows: (i) male or female >18 years of age, and (ii) receiving HD three times a week for at least 6 months. The exclusion criteria were as follows: (i) cardiac implant or pacemaker, (ii) fever, (iii) active malignancy, diarrhea, and polycythemia vera. Ethical approval was granted by the Ethics Committee of Lishui Central Hospital (Approval No. 2022-02) and written, informed consent was obtained from all participants.

#### 2.1.2. Clinical and demographic data

Clinical and demographic characteristics included sex, age, dialysis duration, blood pressure, Kt/V urea dialysis, body mass index (BMI), vascular access, status of diabetes, heart disease, hypertension, levels of N-Terminal Pro-Brain Natriuretic Peptide (NT-ProBNP), serum phosphorus, calcium, parathyroid hormone (PTH), hemoglobin (Hb), and albumin (Alb).

#### 2.1.3. Plasma volume equations

Serum samples were collected for routine blood examinations before and after dialysis. plasma volume was calculated using three previously reported equations: aPV = (1-hematocrit)×(a+(b × weight in kg)) with values of *a* = 864 for females, *a* = 1530 for males, *b* = 47.9 for females and *b* = 41 for males [16]; ideal PV (iPV) = k × weight (kg) with values of *k* = 40 for females and *k* = 39 for males [17]; PVS = ((aPV-iPV)/iPV) ×100%, representing the index of deviation of aPV from iPV [9].

#### 2.1.4. Bioelectrical impedance analysis

BIA was performed concurrently with routine pre-dialysis blood examination using a segmental multifrequency BIA device (BCM, Fresenius, Germany). The parameters measured were total body water (TBW), intracellular water (ICW), extracellular water (ECW), and overhydration (OH). Volume overload was defined as OH/ECW ≥ 15%, as previously described [18,19].

### 2.2. Retrospective cohort

#### 2.2.1. Patients enrollment

To further evaluate the relationship between calculated PVS and outcomes in HD patients, a retrospective study was performed. Patients receiving HD at our hospital between January 2013 and June 2021 were retrospectively enrolled. The inclusion criteria were as follows: i) age >18 years and ii) receiving HD for at least 12 months. Ethical approval was granted by the Ethics Committee of Lishui Central Hospital (Approval No. 2022-02) and written, informed consent was obtained from all participants. The follow-up deadline was June 2022 and the endpoint was all-cause mortality.

#### 2.2.2. Data collection and definition

Data, including sex, age, dialysis duration, Kt/V urea-dialysis, vascular access, status of diabetes, heart disease, hypertension, and levels of serum phosphorus, calcium, PTH, Hb, HCT, and Alb, were retrieved from medical records. The PVS was calculated using HCT. Time-averaged PVS values were also calculated as the mean of the individual PVS values obtained at regular monthly intervals within the first year of dialysis. The Charlson comorbidity index (CCI) encompassing patient age and 16 disease conditions was calculated [14]. The endpoint of the study was all-cause mortality.

### 2.3. Statistical analyses

Variables were presented as mean ± standard deviation, proportion, or median with interquartile range (25%, 75%). Differences between patient groups were compared using an independent-sample t-test or the chi-square test. The Pearson’s correlation test was used to assess the strength of the association between aPV/PSV, BIA, and other parameters. A receiver operating characteristic (ROC) curve was constructed and used to evaluate the diagnostic value of aPV/PVS for volume overload. Kaplan-Meier curves and log-rank tests were used to compare event-free survival rates for all-cause mortality. Multivariate Cox regression analysis was performed to identify the significant prognostic factors affecting all-cause mortality. Statistical significance was set at *p* < 0.05. All statistical analyses were performed using the SPSS 22 software (IBM, Cary NC, USA).

### 2.4. Sample size calculation

Sample size calculation was performed using the PASS 2021 software (NCSS, USA). For the correlation test in the prospective cohort, a sample size of 29 was determined to achieve 81% power. This power allows the detection of a difference of −0.5 between the null hypothesis correlation of 0 and the alternative hypothesis correlation of 0.5. The test used a two-sided hypothesis with a significance level of 0.05. In the retrospective cohort, for COX regression analysis, the study determined that with a sample size of 88 and a covariate with a standard deviation of 1.5, the analysis would achieve 80% power at a 0.05 significance level. This level of power enables the detection of a regression coefficient equal to 0.2.

## 3. Results

### 3.1. Prospective cohort

#### 3.1.1. Correlation between calculated PV and BIA

A total of 54 HD patients with mean age of 54.61 years, 61.11% male), average duration of HD therapy of 38.05 months and mean Kt/V of 1.51 were analyzed (Table 1). Mean calculated aPV was 2537 ± 429 mL, iPV 2349 ± 581 mL and PVS 7.98 ± 6.03%. Parameters measured by BIA were TBW = 35170 ± 6301 mL, ICW = 18350 ± 6507 mL, ECW= 16141 ± 3246 mL and OH =1300 ± 550 mL. aPV was significantly associated with TBW, ICW, ECW, and OH (all *p* < 0.001, Table 2), and most strongly with ECW (*r* = 0.83).

**Table 1.** Clinical and demographic characteristics of HD patients (*n* = 54).

Variables	Value
Male n (%)	33 (61.11)
Age (years)	54.61 ± 12.63
Dialysis duration (months)	38.05 (23.45, 70.54)
BMI (kg/m^2^)	22.04 ± 2.83
Hemoglobin (g/l)	112.25 ± 9.54
Albumin (g/l)	36.15 ± 2.94
Calcium (mmol/l)	2.19 ± 0.14
Phosphorus (mmol/l)	1.57 ± 0.36
PTH (pg/ml)	216.15 (184.43, 359.34)
CRP (mg/l)	1.5 0 ± 0.42
Kt/V urea-dialysis	1.51 ± 0.32
Diabetes n (%)	19 (35.18)
^#^Heart disease n (%)	15 (27.78)
Hypertension n(%)	38 (70.37)

HD: hemodialysis; BMI: body mass index; CRP: C-reactive protein; PTH: parathyroid hormone.

Data are shown as the mean ± SD (standard deviation), percentage or median.

^#^Including heart failure, coronary heart disease, cardiomyopathy and severe heart valve disease.

**Table 2.** Correlation between calculated PV and parameters measured by BIA, NT-ProBNP and blood pressure.

	PVS		aPV	
Parameters	r	*p* value	r	*p* value
TBW	−0.074	0.60	0.721	<0.001*
ECW	−0.089	0.52	0.830	<0.001*
ICW	−0.058	0.68	0.504	<0.001*
OH	0.057	0.68	0.588	<0.001*
ECW/TBW	−0.155	0.23	0.494	<0.001*
NT-ProBNP	0.200	0.111	0.128	0.311
Systolic BP	−0.181	0.150	−0.022	0.862
Diastolic BP	−0.170	0.175	0.076	0.548

PV: plasma volume; BIA: bioelectrical impedance analysis; NT-ProBNP:N-Terminal Pro-Brain Natriuretic Peptide; BP: blood pressure; aPV: actual plasma volume; PVS: plasma volume status; TBW: total body water; ECW: extracellular fluid water; ICW: intracellular fluid water; OH: overhydration; r: correlation coefficient.

*p < 0.01.

#### 3.1.2. Diagnostic value of calculated PV in volume overload

**Figure 2.**
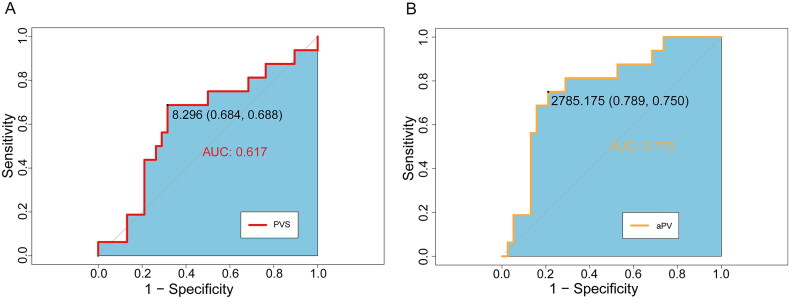
Receiver-operating characteristic (ROC) curve analysis showing the prognostic values of PVS (A) and aPV (B) in predicting volume overload.

The value of aPV and PVS in diagnosing volume overload, defined as OH/ECW ≥15% from BIA measurements, was investigated by plotting an ROC curve (Figure 2A and B). The average OH/ECW was 9.8 ± 8.7%, with 16 (29.6%) patients having OH/ECW ≥15%. The calculated aPV predicted the extent of volume overload, with an AUC of 0.770 (*p* < 0.001). The optimal aPV cutoff value for predicting volume overload was 2785 mL, with sensitivity of 75.0% and specificity of 78.9%. The PVS did not show good diagnostic utility in predicting volume overload (AUC = 0.617, *p* = 0.091).

#### 3.1.3. Correlation between decreased PV and ultrafiltration volume

**Figure 3.**
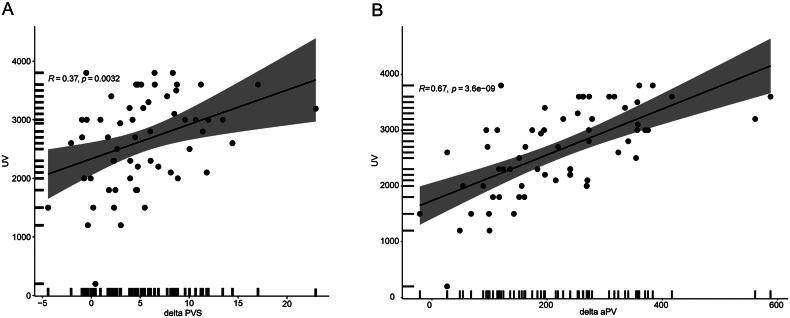
Correlation between ultrafiltration volume and decreased PVS (A) and aPV (B).

Serum samples were collected after dialysis to enable the calculation of aPV and PVS. aPV (2275 ± 578 mL) and PVS (2.68 ± 1.68%) values were both significantly decreased after dialysis (*p* = 0.024 and *p* = 0.009). The mean ultrafiltration volume was 2538 ± 646 mL and mean decreases in aPV and PVS values were 261.88 ± 47.22 mL and 5.32 ± 3.78%. Ultrafiltration volume was significantly associated with decreased aPV (*r* = 0.67, *p* < 0.001) and PVS (*r* = 0.36, *p* = 0.004; Figure 3).

### 3.2. Retrospective cohort: relationship between calculated PVS and outcomes

**Figure 4.**
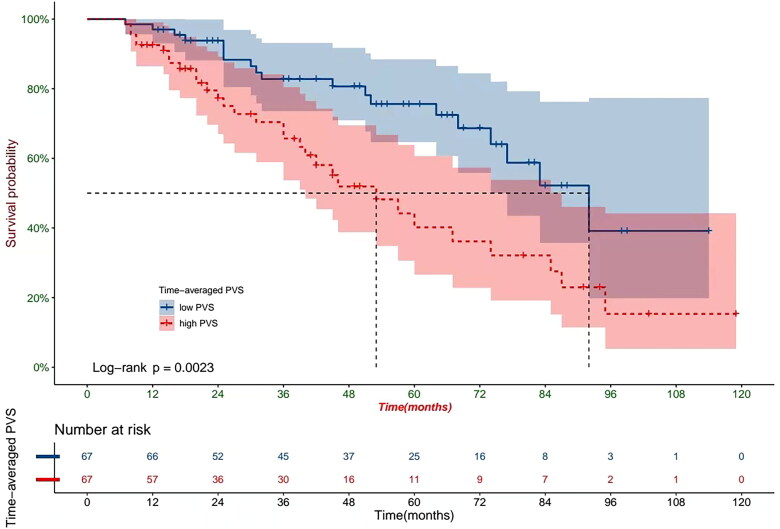
Kaplan-Meier analysis depicting all-cause deaths in HD patients with high and low time-averaged PVS.

The baseline clinical characteristics of the 164 retrospectively enrolled patients on HD are shown in Table 3. The median follow-up time was 53 months (inter-quartile range, 49–67 months; longest follow up, 8 years), during which 60 (36.58%) patients died. The predominant mortality cause was cardiovascular events (51.7%), followed by cerebrovascular accidents (23.3%), infections (11.7%), respiratory failure(5%), and malignant tumor (3.3%). PVS (12.94 ± 10.87% vs. 7.45 ± 5.90%, *p* = 0.024) and time-averaged PVS (12.83 ± 11.20% vs. 6.78 ± 6.22%, *p* < 0.001) values were significantly higher among those patients who died relative to those who survived. Patients who died were significantly older, had higher BUN, FBG, and CCI values, and lower uric acid, TC, and albumin values than those who survived (all *p* < 0.05). COX proportional hazard analysis showed that lower albumin level (HR = 0.912, *p* = 0.020), higher CCI (HR = 1.571, *p* = 0.001), and higher time-averaged PVS (HR = 1.033, *p* = 0.042) were independently associated with mortality (Table 4). Patients were divided into two groups based on a median time-averaged PVS value of 8.72%. A time-averaged PVS value of >8.72% was shown to be significantly associated with increased incidence of all-cause mortality by Kaplan-Meier survival analysis (HR = 2.48, *p* = 0.0023, Figure 4). Besides all-cause mortality, multivariate Cox regression analysis was also performed to identify the prognostic factors affecting cardiovascular mortality. The results showed that higher PVS (HR = 1.063, *p* = 0.024) and higher time-averaged PVS (HR = 1.156, *p* = 0.002) were independently associated with cardiovascular mortality.

**Table 3.** Comparison of surviving and non-surviving HD patients: potential risk factors associated with mortality (*n* = 164).

Variables	Non-surviving patients (*n* = 60)	Surviving patients (*n* = 104)	p value
Age (years)	60.64 ± 8.53	56.42 ± 7.05	0.002*
Male n (%)	33 (55.00)	52 (50.00)	0.537
Duration (months)	45.42(23.51, 71.21)	39.80 (28.42, 69.54)	0.124
Kt/V urea-dialysis	1.51 ± 0.32	1.48 ± 0.61	0.733
Scr (µmol/l)	589.68 ± 192.71	674.30 ± 304.71	0.067
BUN (mmol/l)	18.71 ± 6.42	24.42 ± 8.11	<0.001*
Uric acid (µmol/l)	347.14 ± 96.34	440.81 ± 123.20	<0.001*
WBC (×10^9^/l)	7.01 ± 2.48	6.28 ± 2.34	0.082
Platelets (×10^9^/l)	178.42 ± 82.44	181.89 ± 81.47	0.805
Hemoglobin (g/l)	103.48 ± 24.18	102.84 ± 21.22	0.872
TG (mmol/l)	1.75 ± 1.16	1.86 ± 1.12	0.640
TC (mmol/l)	4.04 ± 1.45	4.72 ± 1.37	0.006*
Albumin (g/l)	33.97 ± 5.82	36.64 ± 5.25	0.004*
FBG (mmol/l)	8.38 ± 5.56	6.67 ± 3.03	0.031*
Calcium (mmol/l)	2.25 ± 0.32	2.14 ± 0.42	0.552
Phosphorus (mmol/l)	1.37 ± 0.47	3.73 ± 0.58	0.407
PTH (pg/ml)	282.88 ± 204.71	327.91 ± 258.44	0.370
Peripheral edema n (%)	10(16.7)	10(9.6)	0.218
^#^Comorbidities n (%)	52(86.70)	79(79.90)	0.110
Hypertension n (%)	38 (63.30)	64 (61.50)	0.819
Diabetes n (%)	30 (50.00)	42 (43.90)	0.232
Heart disease n (%)	39 (65.00)	60 (57.70)	0.357
CCI	7.35 ± 1.32	6.62 ± 1.24	0.002*
PVS (%)	12.94 ± 10.87	7.45 ± 5.90	0.024*
Time-averaged PVS (%)	12.83 ± 11.20	6.78 ± 6.22	<0.001*

HD: hemodialysis; Scr: serum creatinine; BUN: blood urea nitrogen; WBC: white blood cell; TG: triglycerides; TC: total cholesterol; FBG: fasting blood glucose; PTH: Parathyroid hormone; CCI: Charlson comorbidity index; PVS: plasma volume status.

Data are shown as the Mean ± SD (standard deviation), percentage or median.

*p < 0.05.

^#^Including hypertension, diabetes and heart disease.

**Table 4.** Univariate and multivariate Cox proportional hazard analyses for predicting all-cause mortality.

Variables	Univariate analysis			Multivariate analysis		
	HR	95% CI	*p* value	HR	95% CI	*p* value
Age	1.061	1.028–1.101	<0.001*	1.041	0.996–1.091	0.076
Duration	1.022	0.895–1.157	0.007*	1.023	0.938–1.041	0.176
BUN	0.931	0.884–0.957	0.001*	0.983	0.929–1.042	0.585
Uric acid	0.994	0.992–0.99	0.001*	0.999	0.995–1.002	0.415
TC	0.799	0.645–0.99	0.040*	1.052	0.828–1.336	0.669
Albumin	0.861	0.814–0.91	<0.001*	0.912	0.846–0.986	0.020*
CCI	1.407	1.145–1.753	0.001*	1.571	1.199–2.055	0.001*
PVS	1.034	1.019–1.058	<0.001*	1.023	0.993–1.055	0.138
Time-averaged PVS	1.064	1.036–1.091	<0.001*	1.033	1.001–1.065	0.042*

HR: hazard ratio; BUN: blood urea nitrogen; TC: total cholesterol; FBG: fasting blood glucose; CCI: Charlson comorbidity index; PVS: plasma volume status.

*p < 0.05.

## 4. Discussion

In the prospective cohorts, we described the relationship between aPV/PVS and BIA variables, and evaluated the predictive value of aPV/PVS for volume overload in HD patients based on BIA. Volume management is often challenging for HD patients and optimization of the diagnosis and treatment of volume overload remains a priority for nephrologists [20,21]. The relatively free movement of water through the body’s tissues means that, in theory, ICW, ECW, and intravascular water all reflect the volume status. The gold standard for PV measurement is the isotope labeling method [22]. However, BIA quantifies ICW and ECW by measuring the electric current between distant electrodes on the surface of the body and is widely used in HD patients. aPV values, calculated from Hct and weight, were significantly correlated with aPV measured by ^125^Iodine labeling in 119 normal subjects and 30 patients with CHF (*r* = 0.68) [9]. Another study found that TBW measured by BIA showed good agreement with the gold standard deuterium or tritium dilution approaches in 120 healthy individuals and 32 HD patients [23]. The current study found that the calculated aPV was most strongly associated with ECW measured by BIA (*r* = 0.83). The optimal cutoff aPV value for prediction of volume overload was 2785.17 mL and this value had an appropriate sensitivity of 75.0% and specificity of 78.9%, indicating good correlation between calculated PV and BIA.

The calculation of aPV and PVS values demonstrated a significant decrease following dialysis, which was reflected by the ultrafiltration volume. The aPV decreased by 261.88 mL relative to the much larger decrease in the ultrafiltration volume (2275 mL) due to interstitial water flowing back into the blood. PVS value, which indicates relative PV status, decreased from 7.98% to 2.68%. Thus, PVS provides a more accurate reflection of real volume overload than aPV and has been previously utilized to assess pathogenic PV expansion and prognosis [9–15]. However, PVS did not show good diagnostic utility in predicting volume overload in our study (*p* = 0.091), possibly due to BIA itself had limitations for the assessment of volume overload.

In the retrospective cohorts, we mainly evaluated the prognostic utility of PVS in patients undergoing HD. The time-averaged PVS was calculated during the first year of dialysis to accommodate dynamic fluctuations in patient’ dry weight. COX proportional hazard analysis showed that lower albumin level, higher CCI, and higher time-averaged PVS were independently associated with mortality, consistent with previous findings of an association of lower albumin level and higher CCI with mortality in HD patients [24–26]. The hazard ratio (HR) of time-averaged PVS was 1.033, indicating that every 1% increase in time-averaged PVS correlated with an increased risk of all-cause mortality of 3.3%. Dividing the patient cohort by median time-averaged PVS showed that those with a higher time-averaged PVS had 2.48 times higher risk of mortality than those with a lower time-averaged PVS. Ling et al. found a mean PVS of −9% for CHF patients, with a PVS >-4% being associated with increased mortality (HR = 1.65) [9]. Further studies on CHF patients have produced a mean PVS of −11.9% and associated each 5% increment in PVS with a 14% higher risk of all-cause mortality [10]. Evaluation of PVS in the general population by Otaki et al. showed a mean of −2.7% and established that a high PVS of >7% was an independent risk factor for all-cause, cardiovascular, and non-cardiovascular deaths [27]. The current work represents the first examination of the prognostic value of PVS in HD patients, since previous discussions have centered on its utility for patients with CHF. In our study, HD patients exhibited higher PVS values compared to CHF patients in previous studies [9,10]. Additionally, a relatively higher PVS value in HD patients was indicative of a poor prognosis.

The volume in HD patients exhibited dynamic balance, distinct from patients with CHF. Unlike the significance of the initial PVS alone, the time-averaged PVS over the first year proved more indicative of the volume state. In our study, the time-averaged PVS emerged as independently linked to all-cause mortality, whereas the initial PVS did not show the same association. The exact pathophysiological mechanism by which high PVS increases the risk of all-cause mortality remained unclear. One potential explanation was that elevated PVS signaled volume overload, and this overload, stemming from plasma volume expansion, worsened cardiac function [28]. In HD patients, volume overload was linked to hypertension and cardiac dysfunction, emerging as a major risk factor for all-cause and cardiovascular mortality within this population [29].

This study has several limitations. First, the sample sizes were small for both the prospective and retrospective cohorts. Second, BIA was not performed after dialysis, and the relationship between calculated PV and BIA may have been elucidated. Third, further multicenter studies with more patients are required to validate our conclusions. Finally, as BIA data was not available in the retrospective cohort of this study, such a comparison was not possible, further research should investigate whether plasma PV parameters provide a more accurate or less accurate prognosis for mortality compared to BIA parameters.

In summary, the validity of volume overload assessment and the prognostic utility of calculated PV in HD patients have been reported. The calculated aPV was most strongly correlated with ECW measured using BIA, and the optimal cutoff value for predicting volume overload was 2785 mL. HD patients with higher time-averaged PVS of >8.72% had higher rates of all-cause mortality. The present study extends previous findings regarding calculated PV and indicates the potential of PVS as marker to estimate prognosis in patients undergoing HD.
